# Improved acoustic holograms using simulated annealing

**DOI:** 10.1063/5.0258632

**Published:** 2025-04-15

**Authors:** Gagana Weerasinghe, Bram Servais, Daniel Heath, Samuel T. Martin, David J. Collins

**Affiliations:** 1 Department of Biomedical Engineering, The University of Melbourne, Parkville, Victoria, Australia; 2 The Graeme Clark Institute, The University of Melbourne, Parkville, Victoria, Australia; 3 CSIRO Manufacturing, Private Bag 10, Clayton South, Victoria 3169, Australia

## Abstract

Acoustic holography offers the ability to generate designed acoustic fields, enhancing the versatility of acoustic micromanipulation. However, the quality of the generated holograms depends on the nature of the iterative algorithm that is utilized, where the iterative angular spectrum approach (IASA) has been the standard method to date. Here, we introduce a novel approach that categorically improves IASA performance, where we apply the principles of simulated annealing for the generation of high-quality acoustic holograms. We utilize this to realize significant improvements in hologram quality via simulations, fabricated holograms, experimental particle patterning, and high-resolution 2D hydrophone scans. Comparing holograms produced from IASA and/or simulated annealing, we demonstrate that the use of simulated annealing in acoustic holography results in sharper reconstructions and improved hologram outputs across a range of evaluation metrics.

## I. INTRODUCTION

Acoustic holography is an emerging technology that offers unique capabilities in generating designed acoustic fields. Holographic methods, including those based on optical and acoustic fields, form the basis for such applications as volumetric displays,^1^ high density storage systems, and optical^2^ or acoustic tweezers.^3,4^ At the core of holography is a means to store the phase or amplitude profile of the desired wavefront^5,6^ in a way that allows it to be reconstructed by interference when the hologram is illuminated with a coherent source.^5,6^ Modern computer-generated holography (CGH)^7^ removes the need to record a hologram from a physical scene and instead calculates the required phase profile before rendering it for reconstruction. In acoustic holography, designed acoustic fields are generated by modulating the interference of wavefronts in free space with the use of amplitude or phase modulation,^8–11^ where this is emerging as a promising non-contact micromanipulation method and non-invasive energy delivery tool for biomedical applications. Accordingly, acoustic holography demonstrated utility in the aggregation and manipulation of nonorganic particles or even organisms^12–16^ for applications in cell/tissue engineering.^17–19^ Moreover, it has potential applications for neuromodulation,^20,21^ ultrasonic power transfer,^22,23^ cavitation,^24^ mid-air haptics,^25^ ultrasound imaging^26,27^ and particle detection,^28^ where acoustic fields can generate rapid microparticle and cell motion via the emergence of time-averaged pressure and velocity fields.^29–31^

Acoustic holography can be achieved by a variety of approaches to generate the required phase/amplitude maps, including phased array transducers (PATs),^32,33^ in which many ultrasonic devices are individually actuated with varying phase/amplitude. However, the aperture size and drive circuitry of PATs add cost and complexity as the element number increases,^34^ limiting the spatial resolution at which PATs can create sophisticated wavefront manipulation. This accordingly limits the application of PATs for acoustic holography, especially for complex patterns and high-frequency actuation, which may require tens of thousands of microscale, individually addressable transducers to generate high-resolution acoustic fields.

Alternatively, acoustic holography can be achieved via 3D meta-surface plates in which a designed, often static, structure varies the phase/amplitude of propagating wavefronts.^33^ Compared to other acoustic manipulation approaches, acoustic holograms can readily produce unique particle patterns beyond the nodal lines and grids that are otherwise generated in acoustofluidic devices.^35,36^ Additionally, while select acoustic approaches can generate non-periodic patterns,^37,38^ acoustic holograms have the advantage of decoupling structures that modify the acoustic field from the region they are utilized, making them more widely applicable beyond microfluidic devices. A hologram that reconstructs a desired acoustic field can be directly obtained via computer-generated holography instead of recording the phase and amplitude from a physical scene.^12^ Since hologram generation, however, is based on an ill-posed nonconvex inverse problem, which leads to many possible solutions, the performance of the hologram generation algorithm is important to achieve high-quality reconstruction of the acoustic field. Typically, the acoustic hologram encodes the phase information in a single 2D plate, whereafter a coupled wave then forms a pressure pattern in a 3D volume.^12^ Unlike PATs that require an individual transducer to control each pixel, such a fixed microfabricated or 3D printed acoustic hologram offers a simple way to realize a large number of pixels with a single planar transducer. Additionally, such acoustic holograms have the ability not just encoding one target acoustic pattern but also the capability to encode multiple patterns,^12^ enabling programable holograms using only a single hologram,^39^ including for microscale applications.^40^ Conventionally, an iterative angular spectrum approach (IASA),^41^ related to the Gerchberg–Saxton algorithm, has been used for the design of acoustic holograms.^12, 33^ Despite its advantages, IASA has demonstrated performance limitations, leading to various studies proposing enhanced methods. These include phase and amplitude modulation,^10^ iterative backpropagation,^42^ and the weighted Gerchberg–Saxton algorithm.^43^ Recent work has also investigated the application of gradient descent optimization in holograms,^44^ although challenges such as slow convergence rates and the risk of settling in local minima persist. Furthermore, data-driven techniques, including machine learning and deep learning, have been employed in acoustics to optimize amplitude and phase profiles.^45–47^ While these latter methods demonstrate promising improvements in computational efficiency, they are heavily reliant on training datasets and sensitive to initial conditions. Additionally, such approaches impose a substantial computational overhead.

Nevertheless, the optimization of acoustic holograms is crucial for achieving the high-quality reproduction of the targeted patterns. The optimization challenge in holography, however, is formidable due to the multitude of factors influencing hologram quality. Simulated Annealing (SA) has the potential to excel in this complex environment by allowing the exploration of the solution space with a blend of global exploration and local refinement. SA is a probabilistic optimization algorithm inspired by the annealing process in metallurgy, where a material is heated and gradually cooled to reduce defects and enhance its structural integrity.^48^ The algorithm mimics the annealing process by iteratively accepting or rejecting potential solutions based on a probabilistic criterion. At the outset, the system is allowed to explore a broad range of configurations, whereas the algorithm progresses, it gradually reduces the exploration scope and converges toward optimal solutions. By employing controlled randomness and acceptance criteria, the algorithm explores the solution spaces widely, decreasing the likelihood of being trapped in local optima and ensuring a more exhaustive search for a more globally optimal solution.^49,50^ In simulating the cooling process, the SA algorithm introduces the concept of a cooling schedule, which regulates the exploration–exploitation balance during the optimization process. While SA has been applied in optical contexts to the production of a phase-only Fourier computer-generated hologram (CGH)^51^ and a binary computer-generated hologram (BCGH),^52^ the implications of SA for acoustic holography have yet to be explored. As SA provides a more robust and efficient optimization framework by escaping local minima and improving phase stability, it accordingly has the potential to provide meaningful improvements that build on the IASA framework.

**FIG. 1.**
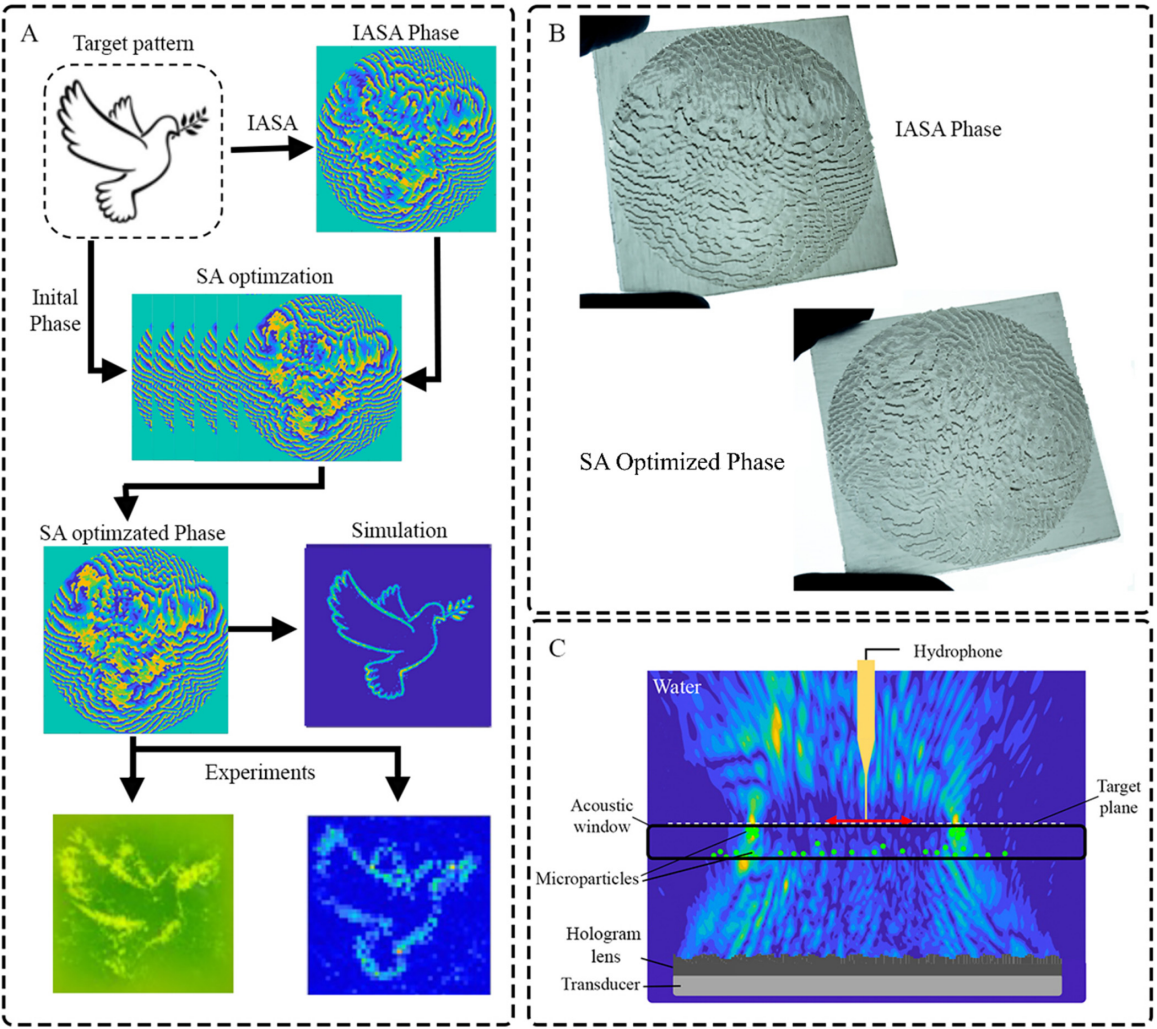
Simulated Annealing (SA) to enhance acoustic holography. (a) Workflow for simulated annealing optimization for the generation of acoustic holograms. (b) 3D printed hologram lenses (held between gloved fingers). (c) Schematic description of acoustic hologram.

Accordingly, we propose a new approach for acoustic holography wherein the principles of SA are applied for the generation and optimization of high-quality acoustic holograms. We investigate two SA implementations, namely, for the improvement of holograms produced via IASA as an initial condition (IASA → SA), as well as the use of SA embedded within the IASA method itself (IASA + SA) [Fig. 1(a)]. Here, we demonstrate that both SA implementations offer the ability to explore a fuller solution space in finding the global optimal solution than IASA alone to create high-quality acoustic holograms.

## II. METHOD AND MATERIALS

### A. Acoustic holography

Acoustic holography can be accomplished by amplitude or phase modulation, where the bulk of prior work utilizes a phase modulation scheme as this allows the use of readily-fabricated 3D phase plates (hologram lenses). In this regard, we consider a single-element ultrasound transducer with a 3D printed holographic lens as a hologram device. A diagram of the experimental setup for acoustic holography is illustrated in Fig. 1(c). This shows the principles of wave propagation and the visualization of the pressure field at the target plane. Here, acoustic waves generated by piezoceramic transducer are modulated by the hologram and pass through a coupling layer (water) into the acoustic window. When the sound wave from the transducer transmits through the hologram, the wave locally experiences a phase delay according to the thickness of the hologram in the propagation direction. The diffracted wave then forms a target image at the image plane in the form of a pressure gradient. Polydimethyl-siloxane (PDMS) particles can then be used to visualize the acoustic field. The acoustic waves modulated by the hologram pass through the fluid into the acoustic window, where these microparticles are pushed up and concentrated at the top of the acoustic window (along +z).^53,54^ Microparticles in the image plane are constrained in their +z movement by the top film, where PDMS microparticles have a negative acoustic contrast and, therefore, move along acoustic force potential gradients toward regions with the highest acoustic field intensity, generating a particle pattern matching that of the target image.^54^

### B. Simulated annealing

Here, we demonstrate the use of SA for generating an improved hologram from an input image. Some of the components used in IASA, including the Angular Spectrum Method (ASM) and forward propagation approach, are adopted into the workflow to simulate the wave propagation and the pressure distribution of the target plane. The use of SA is evaluated here in two implementations. The first implementation we term is the SA-embedded IASA approach (IASA + SA) [Fig. 2(B) in the supplementary material], which uses SA optimization with a random initial condition embedded within the IASA algorithm. The second implementation uses an IASA-generated phase as the initial condition, whereafter an SA workflow is used to generate an improved hologram (IASA → SA) [Fig. 2(A) in the supplementary material]. IASA itself is an iterative method utilizing back and forward propagation, using ASM with a Fourier transform to create the hologram phase. The essential conceit of SA is that the phases from a given iteration are varied randomly, where variations that improve the hologram according to an objective function are chosen for the next iteration. An important feature of the SA algorithm, however, is that it periodically allows the search to move to a new state that is “worse” than the present one in the high-temperature stage. This mechanism prevents the search from being trapped in a local maximum. The probability of accepting poor solutions decreases as temperature is decreased according to the Boltzmann distribution and the algorithm finally converges to the optimum solution.

In the case of IASA → SA, an initial acoustic hologram phase distribution is generated using the IASA method, which is subsequently optimized using SA. Here, the initial phase distribution is generated using 150 IASA iterations for creating the initial hologram phase. Notably, while increasing the number of IASA loops has a large impact on the hologram fidelity for the initial IASA hologram phase, the subsequent application of SA results in uniformly improved hologram quality that is insensitive to the initial number of loops utilized (Table 1 in the supplementary material). Using this IASA-generated hologram phase, the SA optimization initially starts with a higher temperature (T), which mimics heating the entire phase map, where this temperature is subsequently decreased throughout the optimization process. However, if the initial T is too high, it causes prolonged randomness and slows convergence, while if T is too low, it leads to premature freezing, limiting exploration. For this study, the initial T was set to 0.8 based on the scaling and range of the loss function [Eq. (1)], which was found to ensure sufficient flexibility in early iterations while enabling subsequent pixel-level refinements. To apply SA, a randomly chosen area of 20 × 20 pixels of the current phase distribution is selected and then randomized and added to the phase map (Fig. 3 in the supplementary material). Based on this newly randomized neighbor phase, the properties of the resulting acoustic hologram, such as its thickness and transmission coefficient, are calculated. To create the simulated target plane that incorporates the impact from this newly randomized region, the phase is then converted into an angular spectrum via a Fourier transform and propagated to the image plane along the +z axis (forward propagation) by multiplying with the transfer function, with an inverse Fourier transform utilized to generate the target plane pressure field. To evaluate these images, a loss function, which is based on the normalized mean square error (MSE) and the structural similarity index measure (SSIM), is used with

$$\mathrm{Loss}=\frac{\mathrm{MSE}}{\mathrm{SSIM}},$$ (1)

$$\mathrm{MSE}(X,Y)=\;\frac{1}{MN}\underset{m}{\sum }\underset{n}{\sum }{\left[X(m,n)-Y(m,n)\right]}^{2},\;$$ (2)

$$\mathrm{SSIM}(X,Y)=\;\frac{\left(2\mu_{x}\mu_{y}+C_{1})(2\sigma_{xy}+C_{2}\right)}{\left(\mu_{x}^{2}+\mu_{y}^{2}+C_{1})(\sigma_{x}^{2}+\sigma_{y}^{2}+C_{2}\right)}.$$ (3)

These can then be calculated for the generated target plane [Y(m,n)] compared to the desired target input image [X(m,n)]. Here, μ and σ are the mean and variance for each target plane (Y) and for the input image (X), respectively. The revised loss calculated for the newly generated target plane (Loss_new_) can then be compared to the calculated loss for the target plane of the current phase (Loss_current_) as per

$$\Delta E=\mathrm{Los}s_{\mathrm{new}}-\mathrm{Los}s_{\mathrm{current}}.$$ (4)

If Δ*E* < 0, this means that the generated neighbor phase improved the hologram according to the loss function. In this implementation of SA, the changes in Δ*E* < 0 always result in the new phase distribution replacing the current phase distribution in the next iteration of the algorithm. However, if Δ*E* > 0, the new phase is selected according to the following probability function:

$$P=\exp\left(\frac{-\Delta E}{T}\right),$$ (5)

where *T* is the current temperature of the algorithm. The above probability will be compared with a random number 0 < *γ* < 1 generated with a uniform distribution. A tentative solution is accepted when the probability function *P* is greater than the random number *γ*; otherwise, the solution is rejected, as per

$$\{\begin{array}{c} P\left(\frac{-\Delta E}{T}\right)>\gamma \;,\mathrm{accepted},\;\\ P\left(\frac{-\Delta E}{T}\right)<\gamma \;,\mathrm{rejected}.\; \end{array}$$ (6)

This probabilistic criterion [Eq. (6)] is the key step that allows less-optimal phase distributions to be chosen, which helps prevent the algorithm from being trapped in local energy minima. Once a phase distribution has been chosen, the temperature is decreased according to the annealing function. After comparing it with the current hologram, the newly selected current hologram is then compared with the selected best phase hologram using the previously mentioned loss function. If the generated hologram's pressure distribution loss is less than the selected best phase hologram, the current phase (generated phase) is selected as the best optimized phase (Fig. 1 in the supplementary material). The optimization process is performed until the considered temperature (*T*) is sufficiently low. The temperature decreases according to the annealing function:

$$T=\frac{T_{0}}{1+t}\;.$$ (7)

The SA algorithm stops when the temperature reaches $T_{\mathrm{low}}$. After selecting the best phase as the final optimized phase distribution, the phase in the complex pressure field of the hologram plane is converted to a 3D phase hologram model by

$$H_{p}=\frac{\Delta \varphi }{\left(k_{m}-k_{h}\right)}+H_{0},\;$$ (8)

where *H_p_* is the thickness of the pixel and Δ*φ* is the phase shift. *H*_0_ = 1 mm is the substrate thickness and Δ*φ* is set to zero at *H*_0_. The parameters *k_m_* and *k_h_* are the wavenumbers in the medium (water) and the hologram material, respectively.

In the case of the SA-embedded IASA (IASA + SA) method for generating holograms, this follows the same general workflow as with the IASA → SA method, except rather than performing the SA optimization on an initial IASA-generated phase; this starts the generation with a random initial phase profile [Fig. 2(B) in the supplementary material]. The IASA + SA method then varies the phases, utilizing the same forward and backpropagation component of the ASM to determine the pressure field at the target plane from a given phase map, but instead of iteratively looping between the phase and the target plane, as in the IASA, this method generates the phase map using the previously described randomization and selection criteria with the use of the probability and error function. Here we initially set the phase as the random initial phase and generate the target plane by generating the angular spectrum from the initial phase performing forward propagation along the +z axis and then use an inverse Fourier transform to create a pressure pattern at the target plane. We then subsequently compare the target plane pressure with the targeted image selected as a current phase and the current target plane. We then reset the amplitude of the target plane to match the target values while retaining the forward-propagated phase; this maintains the desired target pattern in the algorithm while having the propagated phase for optimization. This is then propagated back to the hologram plane along the −z axis using the ASM with an inverse Fourier transform. From this phase distribution, which consists of 250 × 250 pixels, we then select an area of 20 × 20 pixels in a random region from the phase, randomize that selected area, and replace it in the backpropagated phase (Fig. 3 in the supplementary material), whereafter its thickness and transmission coefficient are calculated. This modulated phase is then considered the neighbor phase of the initial/current phase. We then perform forward propagation and compare the target plane to the target image, with the same loss function and the selection criteria used in the IASA → SA method motioned above. This process is depicted in Fig. 1 in the supplementary material. This process is then continuously iterated until the temperature reaches $T_{\mathrm{low}}$, with an example phase map evolution (for the “dove” image) over successive algorithm iterations in Video 1 in the supplementary material. In both IASA + SA and IASA → SA approaches, the thickness map, comprising the thickness of each pixel (*H_p_*) in the 250 × 250 pixel array, is exported as a 3D STL file using a MATLAB script to subsequently create the physical printed acoustic hologram.

### C. Experimental section

#### 1. Hologram lens fabrication and experiment setup

The hologram 3D files (.STL format) were printed using a 3D printer (Form 3+, Formlabs, Somerville, USA) with UV-sensitive resin [density of 1184 kg m^−3^ and sound speed of 2400 ms^−1^ (Ref. 55)] (UV Sensitive Resin Basic, Formlabs, Somerville, USA). Each hologram consists of 250 × 250 pixels with a pixel dimension of 200 × 200 *μ*m^2^. The washed and cured (Form Wash and Cure Machine, Formlabs, Somerville, USA) holograms were then placed on the surface of a 2.26 MHz PZT ceramic transducer (H4P502000, Huajingda Electronic, Guangdong, China). To avoid cavity formation due to the hologram's microstructure, isopropanol was applied to the hologram surface before water immersion, increasing hydrophilicity and preventing bubbles. The ceramic transducer was wired to a power amplifier (TVA-R5-13A+, Mini-Circuits, Brooklyn, New York, USA) and function generator (AFG 31252, Tektronix, OR, USA) and driven by a sine wave amplified to ≈5 W. The acoustic windows were printed (using the same 3D printer above) and sealed with polyethylene terephthalate (PET) film on both sides to minimize acoustic impedance mismatch, in which the suspension containing PDMS microparticles was encapsulated in the acoustic window. These suspensions were injected into the channel through the syringes and silicon tubes. For the generation of the holograms and for the experiments, the experiments were performed utilizing water (density of 997 kg m^−3^ and sound speed of 1480 ms^−1^).

Hydrophone scanning was performed with the same setup, albeit with the removal of the acoustic windows to allow the introduction of the scanning hydrophone (Precision Acoustics, Dorchester, UK), which consists of a 200 *μ*m hydrophone needle connected to a digital oscilloscope (PicoScope 2000, Pico Technology, UK) to acquire the pressure field. The hydrophone needle is set up and focused on the hologram's target plane [Fig. 1(c)] and the needle translated along both X and Y axes with a step size of 250 *μ*m to perform scanning on the target plane (Fig. 4 in the supplementary material). This is achieved by mounting the hydrophone to a motorized 3D positioning system, with a digital oscilloscope used to acquire pressure readouts. The collected pressure data are then processed to generate a 2D pressure map.

#### 2. Fabrication of PDMS microparticles

The same method used in Ref. 39 was used to create the PDMS microparticles for this study. To prepare the PDMS mixture for the microparticles, a 10:1 mixing ratio of PDMS (Sylgard 184, Dow Corning, MI, USA) was combined with approximately 2 wt. % silicone pigment (Orange Silicone Pigment, Barnes, NSW, Australia). For fluorescent particles, we added fluorescent dye to the PDMS mixture and mixed carefully. This mixture was blended with a water solution containing 1 wt. % surfactant (Pluronic F-127, Sigma-Aldrich, MO, USA). The resulting PDMS mixture was then homogenized (Ultra-Turrax T-25, IKA, Staufen, Germany) for 20 min with a speed of 17 000 rpm at room temperature. After homogenizing, it was then stirred at 40 °C for 4 h. Following this, it was left to cure for a minimum of 24 h at room temperature. The cured PDMS microparticles were then used in all the experiments.

## III. RESULTS AND DISCUSSION

Here, we compare the performance of acoustic hologram algorithms, noting the improvements that the implementation of SA approaches has over the use of IASA alone. In our numerical studies, we quantitatively evaluate hologram outputs according to objective similarity, error, and signal-to-noise criteria. We further compare the performance via experimental evaluations with particle patterning and high-resolution 2D hydrophone scans. These serve to not just establish the improvements that an SA implementation offers, but also to demonstrate these in physical implementations. The selected patterns represent varying complexity levels and align with previously studied patterns (dove,^12^ bicycle^54^ in acoustic holography), facilitating cross-study comparisons.

### A. Numerical evaluation

The reconstructed acoustic images from the simulations were quantitatively analyzed according to multiple metrics including cosine similarity (CSIM), MSE, and peak signal-to-noise ratio (PSNR), each of which represents a unique measure of the fidelity and a comprehensive evaluation of the hologram compared to the target image. CSIM is a metric used to measure the similarity between two vectors, which calculates the cosine of the angle between two vectors in a multi-dimensional space, which helps in determining how similar they are to each other. This quantifies how similar two patterns are based on their directions, rather than their magnitudes, and accordingly offers insights into structural and directional similarity, which is important in applications such as holograms where the overall pattern is more important than exact pixel values. MSE measures the average of the squares of the differences between corresponding pixel values in the original and generated images, giving a measure of pixel-wise differences. This, therefore, gives a straightforward measure of pixel-level differences, highlighting large discrepancies between pattern magnitudes. Finally, PSNR measures the ratio between the maximum possible power of a signal (original pattern) and the power of corrupting noise (difference between original and generated patterns), serving as a measure of the quality of the generated pattern in terms of signal-to-noise ratio, making this useful for assessing image quality and perceptual similarity. Here, CSIM and MSE assess the ability of the hologram to produce uniform, maximum intensity acoustic fields according to the target image design, whereas PSNR is useful in assessing the speckle noise in the image. The evaluation functions are as follows, with

$$\mathrm{CSIM}=\frac{\sum (A_{\mathrm{target}}\cdot A_{\mathrm{hologram}})}{\sqrt{\sum A_{\mathrm{target}}^{2}\;}\times \sqrt{\sum A_{\mathrm{hologram}}^{2}}},$$ (9)

$$\mathrm{MSE}=\;\frac{1}{MN}\underset{m}{\sum }\underset{n}{\sum }{\left[A_{\mathrm{target}}(m,n)-A_{\mathrm{hologram}}(m,n)\right]}^{2}\;,$$ (10)

$$\mathrm{PSNR}=10\log_{10}\left(\frac{MAX^{2}}{\mathrm{MSE}(A_{\mathrm{target}},A_{\mathrm{hologram}})}\right)\;,$$ (11)

where $A_{\mathrm{target}}$ and $A_{\mathrm{hologram}}$ denote the amplitude of the inputted target image and the pressure field reconstructed from the hologram, respectively , *M* and *N* are the dimensions of the fields, and MAX is the maximum possible pixel value of the image.

**FIG. 2.**
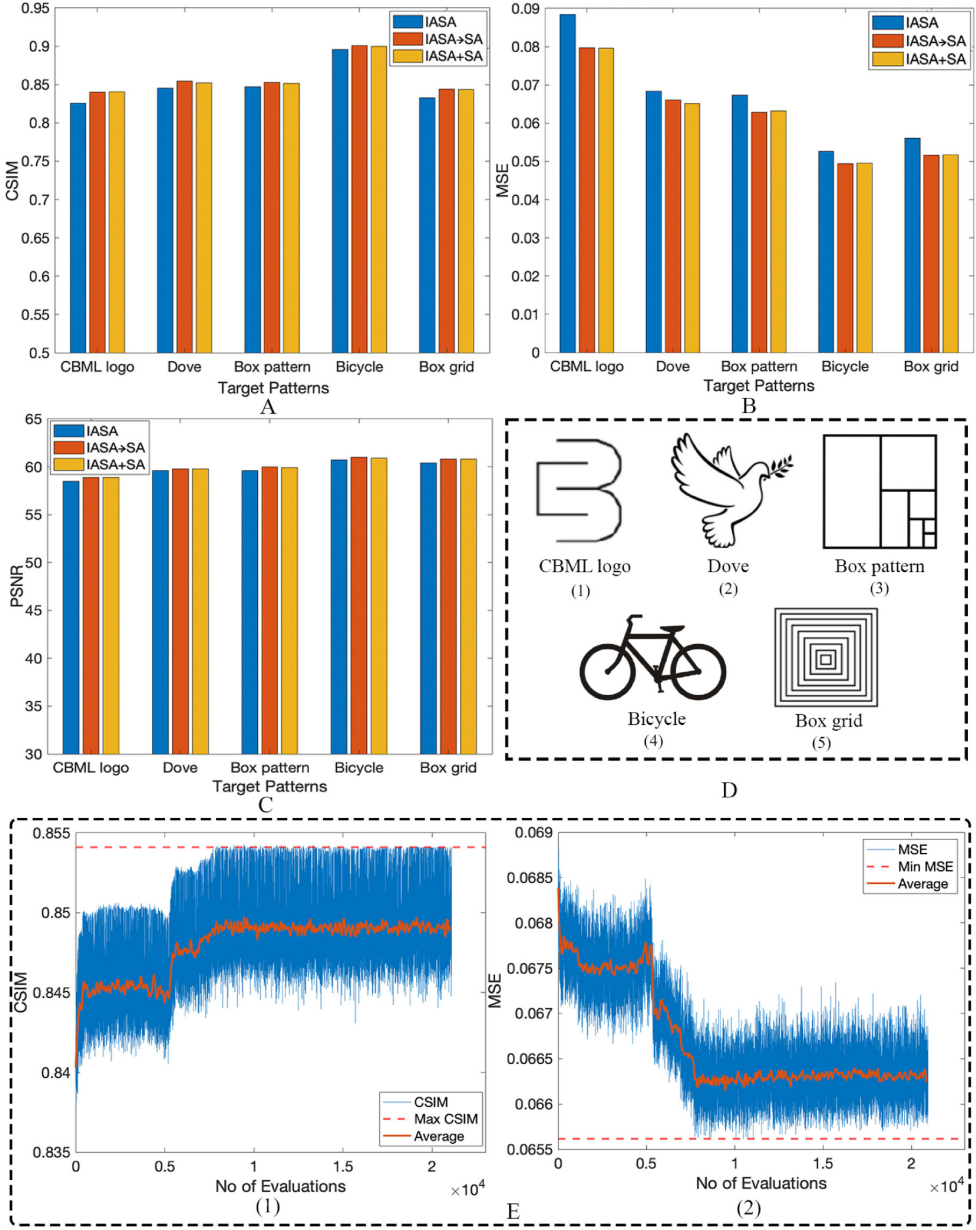
Graphical representation of the numerical evaluation (data from Table I). (a) Cosine Similarity (CSIM), (b) Mean Square Error (MSE), and (c) peak signal-to-noise ratio (PSNR) for IASA, IASA → SA, and IASA + SA. (d) Target patterns used for SA; (1) CBML logo, (2) dove, (3) box pattern, (4) bicycle, (5) box grid. (e) The SA optimization process for dove pattern as evaluated via CSIM and MSE.

Figure 2 quantitatively compares different algorithms, where Figs. 2(a)–2(c) demonstrate that the addition of SA via IASA → SA and IASA + SA approaches uniformly generate more accurate holograms compared to IASA alone, with reductions in the MSE and increases in CSIM scores. Table I highlights the individual data points shown in Fig. 2, where the algorithm exhibiting the best performance for a given target is highlighted. In all cases, IASA → SA or IASA + SA generates improved holograms compared to IASA alone, with minimal variation between SA implementations. While all metrics demonstrate an improvement with the addition of SA, the MSE exhibits a somewhat greater improvement relative to the other metrics, a result attributable to its role in the loss function, where it is explicitly minimized. Its high sensitivity to pixel-wise differences makes it a robust measure of similarity. Figure 2(e) illustrates the change in the CSIM and MSE values as the optimization process progresses for the dove pattern. Specifically, it plots these metrics against the number of evaluation loops performed. This visual representation reflects the working progress detailed in Sec. II B. As the optimization progresses, the algorithm does not simply retain only the best-performing phase configurations. Instead, it also maintains and explores tentative solutions, even those with less-optimal phase distributions. This strategy is crucial for preventing the algorithm from becoming trapped in local minima, allowing it to explore a wider range of potential solutions and find a more globally optimal result.

**TABLE I.** Numerical Evaluation comparison among IASA and SA using CSIM, MSE, and PSNR. Boldface values indicate the best performance for each metric across the three approaches.

Target pattern	CSIM			MSE			PSNR (dB)		
	IASA	IASA → SA	IASA + SA	IASA	IASA → SA	IASA + SA	IASA	IASA → SA	IASA + SA
CBML logo	0.8255	0.8399	**0** **.** **8407**	0.0884	0.0797	**0** **.** **0796**	58.5	58.9	**58** **.** **9**
Dove	0.8453	**0.8543**	0.8522	0.0684	0.0661	**0** **.** **0651**	59.6	**59** **.** **8**	59.8
Box pattern	0.8468	**0** **.** **8529**	0.8518	0.0673	**0** **.** **0628**	0.0632	59.6	**60** **.** **0**	59.9
Bicycle	0.8958	**0** **.** **9011**	0.8995	0.0526	**0** **.** **0494**	0.0495	60.7	**61** **.** **0**	60.9
Box grid	0.8328	**0** **.** **8441**	0.8434	0.0561	**0** **.** **0516**	0.0517	60.4	**60** **.** **8**	60.8

### B. Experimental evaluation

In this section, we visualize the acoustic fields formed by IASA, IASA→SA, and IASA+SA approaches, highlighting the impact of the different SA approaches on both the hologram phase and the acoustic field generated. Fluorescent PDMS particles were used for the experimental visualization of the holographic patterns. All the experiments were performed using a 2.26 MHz actuation frequency and with an amplitude of −8 dBm, where the target plane for all holograms is set to 25 mm from the transducer plane. Hydrophone scanning was also performed to map the pressure variations in the target plane, creating a detailed picture of the sound pressure distribution across a specific plane. This mapping provides valuable insights into the behavior of the sound waves, allowing evaluation across hologram implementations.

**FIG. 3.**
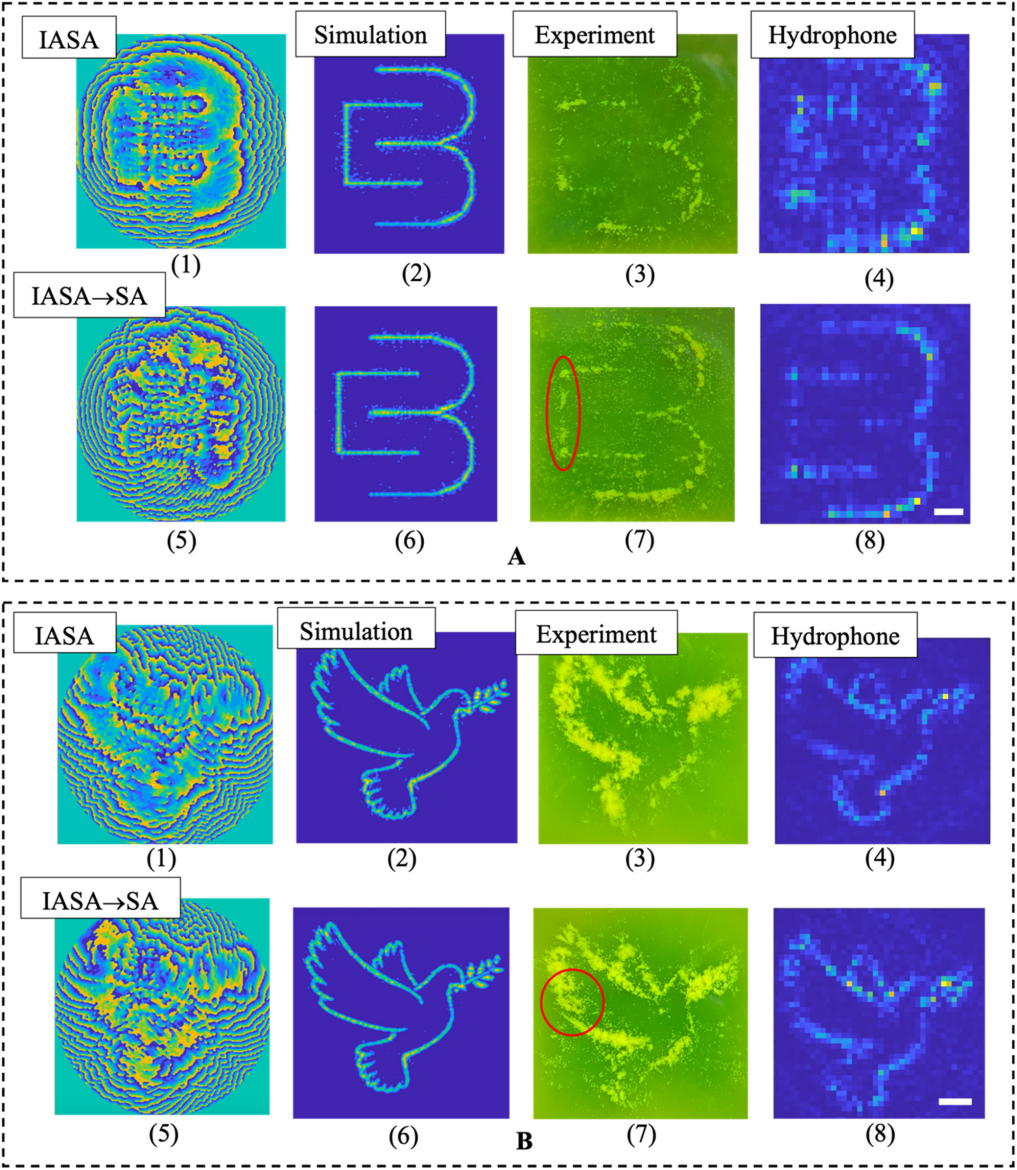
Experimental and simulation results for CBML logo and dove images. [a(1), a(5), b(1), and b(5)[ SA-optimized and IASA-generated acoustic hologram phases. [a(2), a(6), b(2), and b(6) [Simulated target image for SA and IASA. [a(3), a(7), b(3), and b(7)] Experimental particle patterning for SA and IASA. [a(4), a(8), b(4), and b(8)] Hydrophone scan results for SA and IASA.

**FIG. 4.**
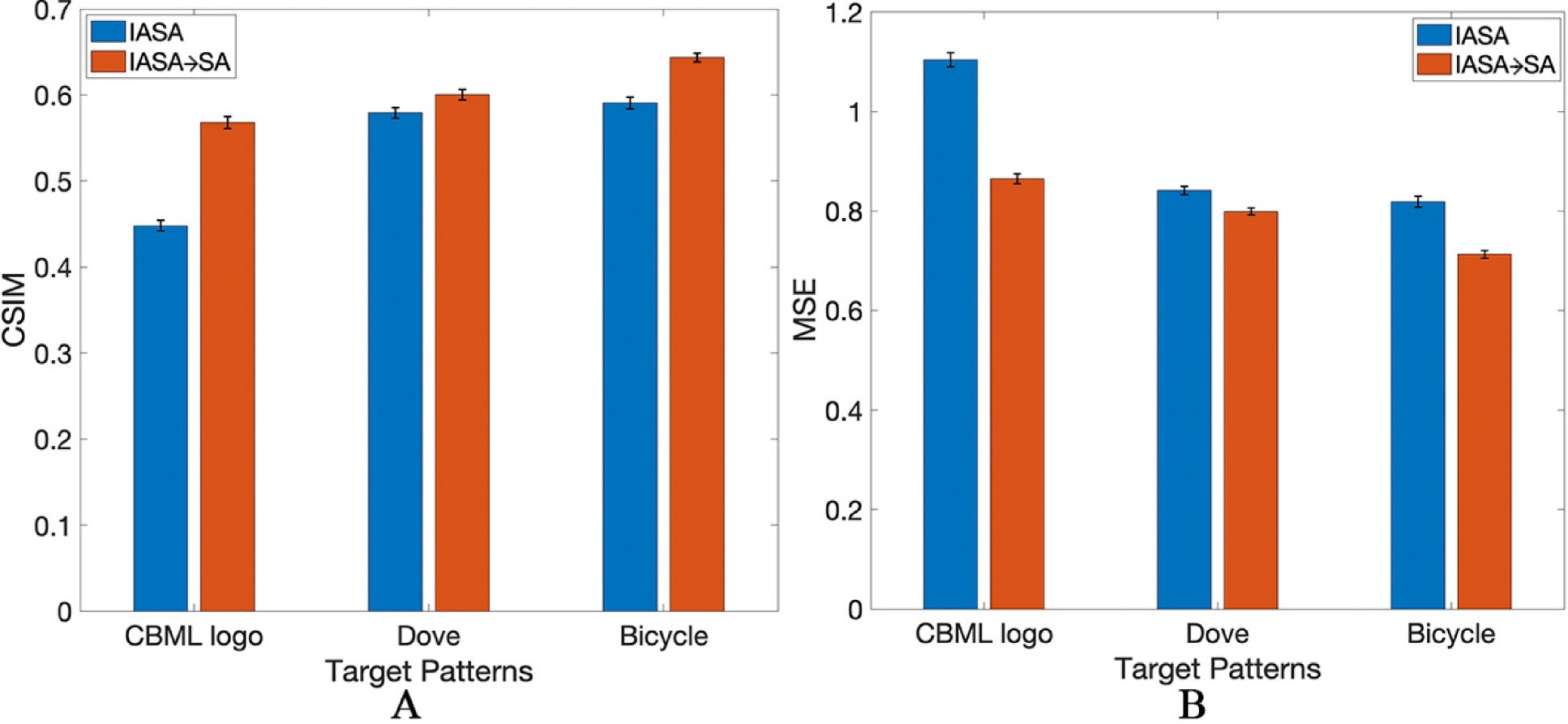
Graphical representation of the quantitative analysis for the hydrophone scans. (a) Cosine Similarity (CSIM) and (b) Mean Square Error (MSE).

To evaluate the difference and improvement of using SA optimization, we first compare the simulation and experimental results between the IASA → SA and IASA-only target patterns generated from the hologram phases (Fig. 3). Figure 3(a), for instance, shows the simulation of a representative image (the CBML lab logo), where the IASA → SA results in less noise and scattering of the target image compared to IASA alone. Figure 3 also includes the experimental results for these different approaches, wherein we note an improved pattern in the IASA → SA case, with enhanced and more uniform focusing. Similar improvements can further be seen from the hydrophone scans. These experiments were carried out for different target patterns (Fig. 3 and Fig. 6 in the supplementary material) where the improvements among the different patterns can be observed both quantitatively (Figs. 2 and 4) and qualitatively in both experimental patterns (Fig. 3 and Fig. 6 in the supplementary material) and numerical evaluations (Fig. 2). This can be further validated from the quantitative analysis performed on the hydrophone scans (Fig. 4) wherein both CSIM and MSE values are shown for this experimental dataset. Accordingly, the IASA → SA approach in reducing spatial variations along the focusing lines appears to reduce diffractive effects associated with the use of a spatially limited transducer, where (for instance) the IASA hydrophone scan in Fig. 3(a) evidences somewhat more periodic amplitude variation than that from the SA-optimized hologram. Comparing the quantitative analysis performed in numerical evaluation [Figs. 2(a) and 2(b)] and the experimental evaluation (Fig. 4), we can observe that experimental results for SA-optimized holograms appear to demonstrate somewhat greater absolute improvements in CSIM and MSE compared to simulation ones, although this can be understood in the context of comparatively greater distortion, noise, and worse performance in experimental settings compared to simulation ones generally, where CSIM and MSE values are uniformly worse for experimental measurements. Accordingly, this apparent discrepancy between the differences observed in simulation vs experimental results can be understood in terms of the proportional improvement approaching a maximum/minimum value from an IASA baseline, rather than the absolute difference in CSIM/MSE quantities. Nevertheless, the overall difference for the “dove” and “bicycle” experimental results (on the order of a 5%–10% difference) is roughly in line with that observed in the simulations. Here, the simulation data lack the full complexity of real-world noise, distortions, or artifacts present in hydrophone scans, where the SA algorithm demonstrates that it is effective in producing robust holograms that do not require experimental fine-tuning to produce improved outcomes in real-world conditions. In further evaluating and comprising the improvements of SA in Fig. 3(a), we can see that the SA-optimized holograms are able to trap more particles compared to IASA-only hologram. In the marked area [Fig. 3a(7)], more complete trapping was achieved compared to the IASA hologram. This is reflected in the hydrophone scan, which shows enhanced and more uniform focusing. The same phenomenon can be observed in Fig. 3(b) and Fig. 6 in the supplementary material as well, where the hydrophone scans demonstrate SA improvements to acoustic focusing, which has led to improved and accurate patterning with particles [Fig. 3b(7) and Fig. 6(C) in the supplementary material].

**FIG. 5.**
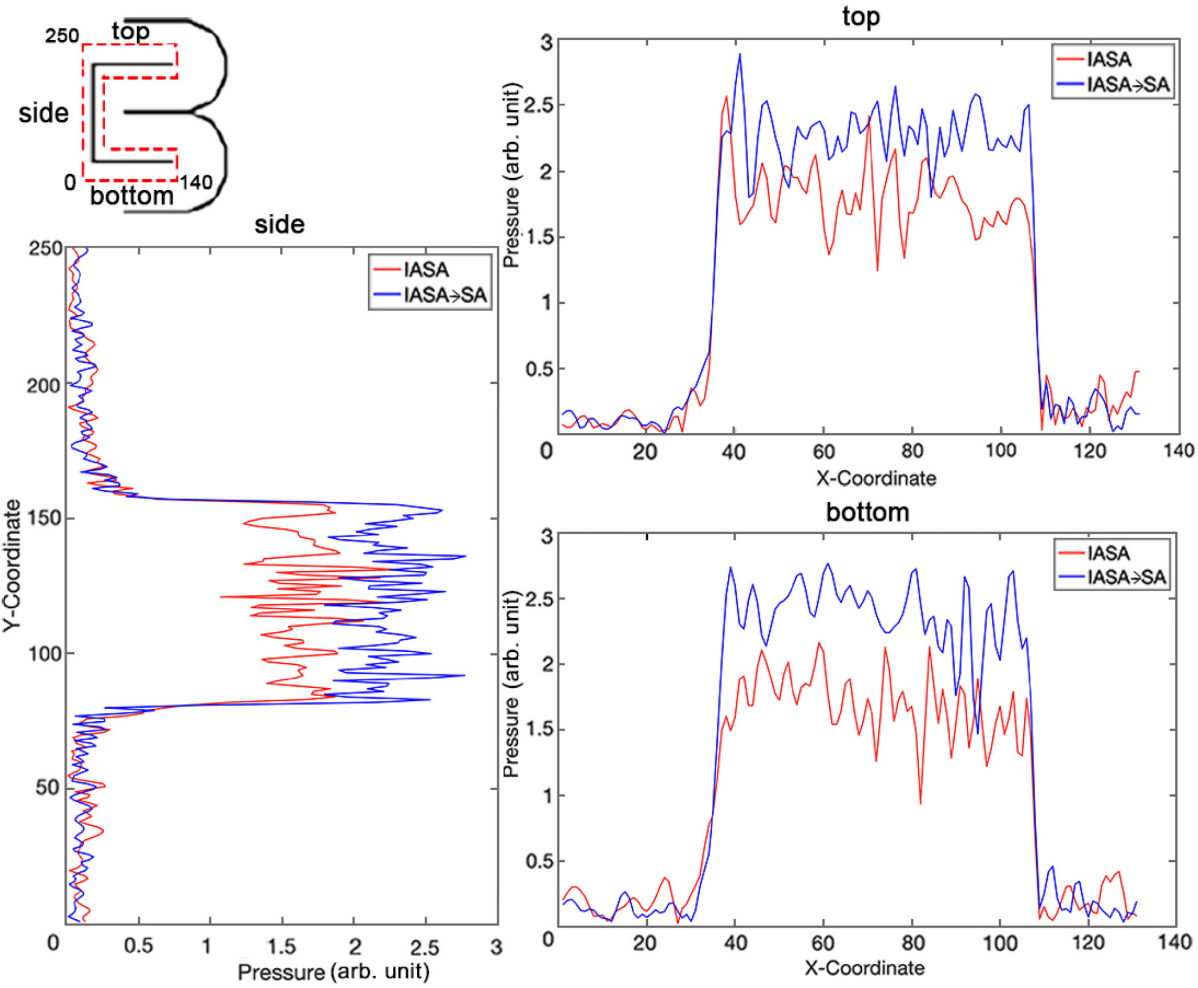
Local comparison of acoustic pressure amplitude in a CBML logo hologram generated via IASA and IASA → SA.

The error bars in Fig. 4 are calculated by first taking hydrophone scans with multiple different pressure measures at the same point within a given hologram. The standard deviation of these adjusted results was then calculated to determine the error bars. We can further quantitatively assess the improvement in the target image uniformity along focusing lines, where Fig. 5 shows the pressure distribution for the CBML logo [from Fig. 3(a)] across a subset of these features. The dashed lines in the simulation image denote the pathlengths along which the pressure amplitude is plotted. We note here that the pressure amplitude is somewhat less variable with IASA → SA, with a smaller differential between the maximum and minimum pressure points, and interestingly consistently greater pressure magnitudes overall. IASA alone yields suboptimal pressure magnitudes due to its local optimization nature, often converging to a local minimum. While it refines the acoustic field, it does not guarantee a global optimum. SA improves this by using stochastic, non-local updates that help escape local minima. SA accepts worse solutions early on to explore a broader range of possibilities and then refines toward a near-global optimum. This enables the generation of SA-optimized phase distributions that maximizes energy concentration in the target area, achieving higher pressure magnitudes and improving wavefront coherence than IASA alone. This results in a more precise acoustic field, where the constructive interference is maximized, and destructive interference is minimized.

**FIG. 6.**
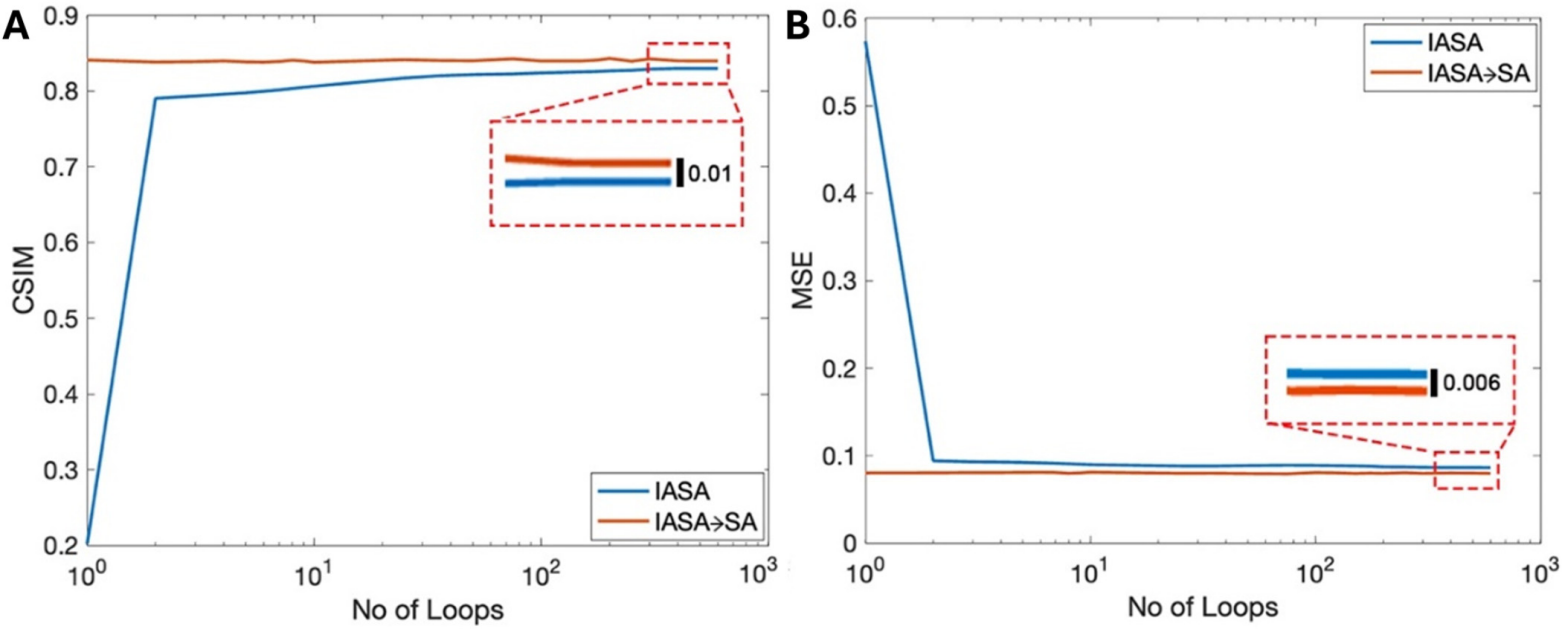
(A) For IASA → SA method CSIM for SA and IASA with respect to the number (no.) of loops used in IASA. (b) MSE distribution for SA and IASA with respect to the no. of loops used in IASA.

As highlighted previously, the SA optimization approach can be used in tandem with IASA, with SA optimization occurring with the use of an IASA-generated initial phase (IASA → SA). To assess any dependency on the IASA-derived hologram quality with the accuracy of the optimized phase, Fig. 6 assesses the CSIM and MSE according to the number of loops that used to create the initial IASA phase, with more IASA iterations generating an improved hologram. Here, the blue line indicates these parameters for the initial IASA phase, with the orange line indicating the corresponding CSIM and MSE after the application of SA optimization. In all cases, SA improves the hologram quality, regardless of the number of IASA loops utilized to create the initial condition. These data alternatively are represented in tabular and bar-chart forms in Table 1 and Fig. 5 in the supplementary material. From these, we can further observe that the number of loops used to create the initial phase does not impact the final optimized results, with essentially identical CSIM and MSE scores post-SA optimization, indicating that SA is independently capable of generating globally optimized results regardless of starting conditions.

**FIG. 7.**
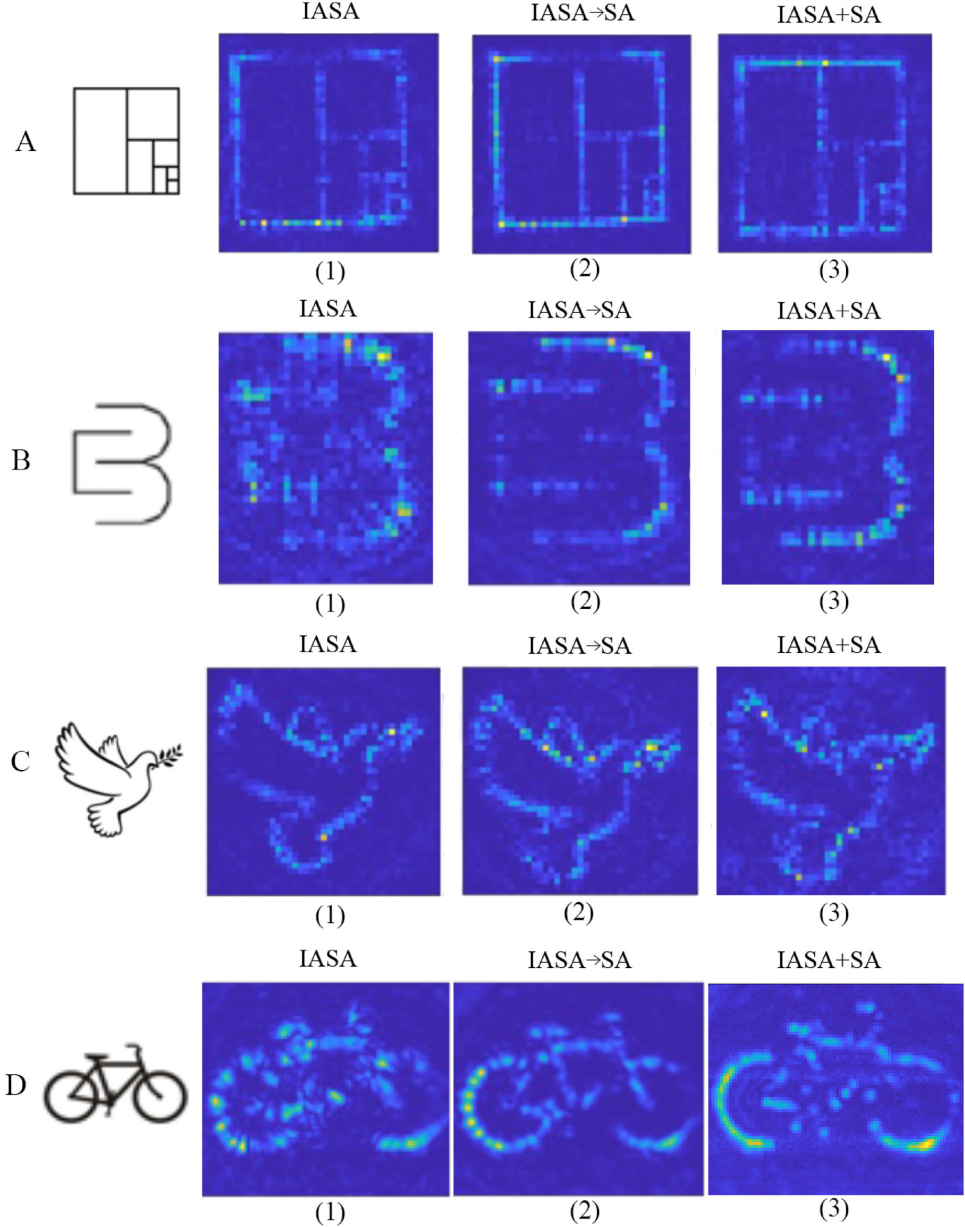
Hydrophone scans for IASA, IASA → SA, and IASA + SA approaches.

We further experimentally demonstrate the ability to generate IASA + SA holograms, where hydrophone scans from IASA, IASA → SA, and IASA + SA holograms are shown in Fig. 7. SA optimization yields generally improved results, as expected from Fig. 2, noting that the SA-optimized hydrophone scans evidence greater uniformity with less periodic effects across the realized pressure amplitudes, especially evident in the case of the CBML logo [Fig. 7(b)] and bicycle [Fig. 7(d)] holograms, which can also be observed in Fig. 4. Here, integrating the SA optimization process enhances hologram generation by addressing the limitations of IASA's local optimization approach. As observed in the marked areas of Fig. 3a(7) and Fig. 6 in the supplementary material, SA produces more refined and smaller features compared to IASA. This improvement stems from SA's ability to introduce intermediate phase patterns, allowing local detail enhancement even when overall accuracy remains low. Unlike other methods that may reject such solutions, SA's probabilistic acceptance criterion enables further refinement, ensuring the formation of continuous structures, such as straight or curved lines. Additionally, through stochastic, non-local updates, SA facilitates the escape from local minima, yielding optimized phase distributions that enhance energy concentration in the target region. This results in higher pressure magnitudes, improved wavefront coherence, and greater control over the acoustic field. Accordingly, SA, enhances all patterns, although the extent of this potential improvement will be due to the relative delta between IASA outcomes and a global optimum, which will be dependent on how well the target pattern may be produced via IASA in the first case. For instance, the CBML logo exhibits a somewhat higher improvement in MSE compared to other shapes, as observed in both simulated (Fig. 2) and experimental (Fig. 4) data. In this case, the combination of straight and curved lines over a large area may lead the CBML logo to be more likely to result in a less-optimal IASA-induced local minima. Nevertheless, the SA framework remains broadly applicable to diverse target patterns, making it a robust and versatile approach for improved holographic design.

## IV. CONCLUSION

Here, we investigate a unique approach based on simulated annealing optimization to produce improved, high-quality acoustic holograms. Our findings demonstrate that simulated annealing can effectively improve the quality of holograms, achieving sharper reconstructions and reducing artifacts. This offers several advantages over previous optimization techniques, including its ability to escape the local minima inherent to the IASA approach and find more globally optimal solutions. Notably, where an IASA-derived phase map is used as the initial condition, SA is able to improve the hologram to an equivalent degree regardless of the IASA-generated quality. Furthermore, embedding SA within the IASA iterations yields similarly improved outcomes. These improvements are demonstrated using both numerical and experimental evaluation methods, evidencing improvement across all test cases with the incorporation of SA. Furthermore, while here we implement SA for the improvement of phase holograms, this could similarly be applied to amplitude-based ones as well. As the SA approach can be implemented in a straightforward way by integrating this with conventional IASA, this has the potential to be implemented across fields where acoustic holography is relevant. This accordingly has the potential to benefit a wide range of applications in which accurate pressure field control is required, including micromanipulation in acoustofluidics, tissue engineering, underwater communication, real-time sensing, and medical ultrasound. Future research directions include exploring more sophisticated annealing schedules and incorporating domain-specific knowledge into the optimization process, including the use of alternative phase and amplitude modulation strategies beyond static 3D printed plates.

## SUPPLEMENTARY MATERIAL

See the supplementary material for the optimization workflow, randomization method, performance metrics, and experimental/simulation results.

## Data Availability

The data that support the findings of this study are available from the corresponding author upon reasonable request.
